# Assessment of Body Condition in African (*Loxodonta africana*) and Asian (*Elephas maximus*) Elephants in North American Zoos and Management Practices Associated with High Body Condition Scores

**DOI:** 10.1371/journal.pone.0155146

**Published:** 2016-07-14

**Authors:** Kari A. Morfeld, Cheryl L. Meehan, Jennifer N. Hogan, Janine L. Brown

**Affiliations:** 1 Lincoln Children’s Zoo, Lincoln, Nebraska, United States of America; 2 Center for Species Survival, Smithsonian Conservation Biology Institute, Smithsonian National Zoological Park, Front Royal, Virginia, United States of America; 3 AWARE Institute, Portland, Oregon, United States of America; University of Florida, UNITED STATES

## Abstract

Obesity has a negative effect on health and welfare of many species, and has been speculated to be a problem for zoo elephants. To address this concern, we assessed the body condition of 240 elephants housed in North American zoos based on a set of standardized photographs using a 5-point Body Condition Score index (1 = thinnest; 5 = fattest). A multi-variable regression analysis was then used to determine how demographic, management, housing, and social factors were associated with an elevated body condition score in 132 African (*Loxodonta africana*) and 108 Asian (*Elephas maximus*) elephants. The highest BCS of 5, suggestive of obesity, was observed in 34% of zoo elephants. In both species, the majority of elephants had elevated BCS, with 74% in the BCS 4 (40%) and 5 (34%) categories. Only 22% of elephants had BCS 3, and less than 5% of the population was assigned the lowest BCS categories (BCS 1 and 2). The strongest multi-variable model demonstrated that staff-directed walking exercise of 14 hours or more per week and highly unpredictable feeding schedules were associated with decreased risk of BCS 4 or 5, while increased diversity in feeding methods and being female was associated with increased risk of BCS 4 or 5. Our data suggest that high body condition is prevalent among North American zoo elephants, and management strategies that help prevent and mitigate obesity may lead to improvements in welfare of zoo elephants.

## Introduction

Obesity is a significant health problem for humans [1–3], companion animals, [4–6] and livestock [7, 8]. Likewise, there is growing concern about the health of zoo animals as it relates to obesity and related conditions [9–12]. In elephants, obesity has been speculated to be a problem because of plausible associations with conditions threatening health and population sustainability, such as cardiovascular disease, arthritis and foot problems, and ovarian cycle abnormalities [10,12,13–19].

Obesity is defined as an accumulation of excessive amounts of adipose tissue (fat) in the body [20]. All measures of adiposity involve defining body composition, or the relative amounts of fat versus lean body mass. Various techniques are available to measure body condition, and these differ in applicability according to the species of interest and the context of the assessment. For example, criteria have been established for what constitutes “overweight” and “obese” in humans, and are usually based on measures of adiposity, such as body mass index (BMI) (weight divided by height^2^) using epidemiological methods. Dogs and cats are classified as overweight when their body weight is >15% above the defined normal for the species, and as obese when their body weight exceeds 30% of the optimal weight [20].

A body condition score (BCS) estimates adiposity based on visual or tactile evaluations of muscle tone and key skeletal elements [20–21]. A number of scoring systems have been developed for a variety of species, and scores are commonly based on an ordinal 5- or 9-point scale [20, 22]. Low scores represent animals with less body fat, whereas higher scores represent animals with more body fat. For example, two numeric scales are typically used and accepted in veterinary practices for assessing body condition in dogs (5-pt and 9-pt scales) [23, 24]. When using a 5-point scale, the “ideal/normal” BCS = 3, BCS = 1–2 equates to “underweight/thin” and “overweight/obese” includes BCS = 4–5. When using a 9-point scale, the “ideal/normal” BCS = 4–5, whereas “underweight/thin” is represented by BCS = 1–3 and “overweight/obese” include BCS = 6–9. Similarly, in cattle, both 9-point [25, 26] and 5-point [27, 28] scales are used, and the middle scores represent the “ideal/normal” distribution of body fat. In some cases, these visual scales were validated using additional biological measures of adiposity. For example, ultrasound measures of actual fat thickness have been used to validate BCS methods in a number of domestic and non-domestic species, including cattle [22], moose (*Alces alces*) [29], elk (*Cervus elaphus*) [30], woodland caribou (*Rangifer tarandus caribou*) [31], pinnepeds [32], and African elephants (*Loxodonta africana*) [19]. Physiological measures of adiposity have also been used for validating visual scales. For example, serum triglycerides, which are stored in adipose tissue and involved in fat deposition [33, 34], were found to correlate with visually assessed body condition scores in tiger sharks [35] and dogs [36]. In addition, leptin, which is synthesized and secreted primarily by adipocytes [37], has been shown to positively correlate with body condition scores in rodents [38], horses [39], and dogs [40, 41]. Body condition scoring systems are routinely used in the management and care of many species, including horses, cattle, sheep, mice, and dogs [42, 43], where BCS at either end of the scale (i.e., very thin or very fat) can indicate compromised welfare. For example, emaciation or low BCS may result from inadequate feed intake, inappropriate nutrition, chronic disease, poor dental care, or parasitism [44, 45]. At the other extreme, high BCS (i.e. obesity), a problem speculated for zoo elephants [10, 12, 13–19], may be a concern due to the host of secondary diseases that can accompany adiposity.

The principal cause of obesity in any species is an energy imbalance, where caloric intake exceeds energetic expenditure. In humans, the risk factors associated with obesity have been thoroughly investigated and include a variety of lifestyle, environmental, and genetic factors [46–48]. Although a number of factors within the zoo environment are likely to influence body condition in elephants, there is a paucity of literature that scientifically investigates elephant obesity and associated risk factors. Therefore, the objectives of this study were to 1) determine the distribution of body condition scores of elephants in accredited North American zoos for the full population and by species and sex, and 2) use multi-variable regression modeling to determine the demographic, management, housing, and social factors associated with increased risk of elephants being classified as overweight or obese.

The 5-point visual index used for assessing body condition in African elephants was previously developed and validated with ultrasound measures of subcutaneous fat by Morfeld et al. [19]. In the present study, a similar BCS index for Asian elephants was developed and tested for inter-assessor reliability and biological validity. Our study is the first large-scale investigation of elephant body condition and was a component of a larger study entitled “Using Science to Understand Zoo Elephant Welfare”, a multi-institutional collaborative effort to produce scientific data to support decision making with regard to best practices in elephant management [49].

## Materials and Methods

### Ethics Statement

All data included in this study were sourced from elephant programs at 65 zoos accredited by the Association of Zoos and Aquariums (AZA) and animals enrolled in the Using Science to Understand Zoo Elephant Welfare study [49]. These zoos were located in the United States, Mexico and Canada. Both zoo-level and elephant-level data were collected. This study was authorized by the management at each participating zoo and, where applicable, was reviewed and approved by zoo research committees. In addition the study protocol was reviewed and approved by the Zoological Society of San Diego Institutional Animal Care and Use Committee N.I.H. Assurance A3675-01; Protocol 11–203.

### Development and Testing of the Asian Elephant Body Condition Scoring Index

**Fig 1:**
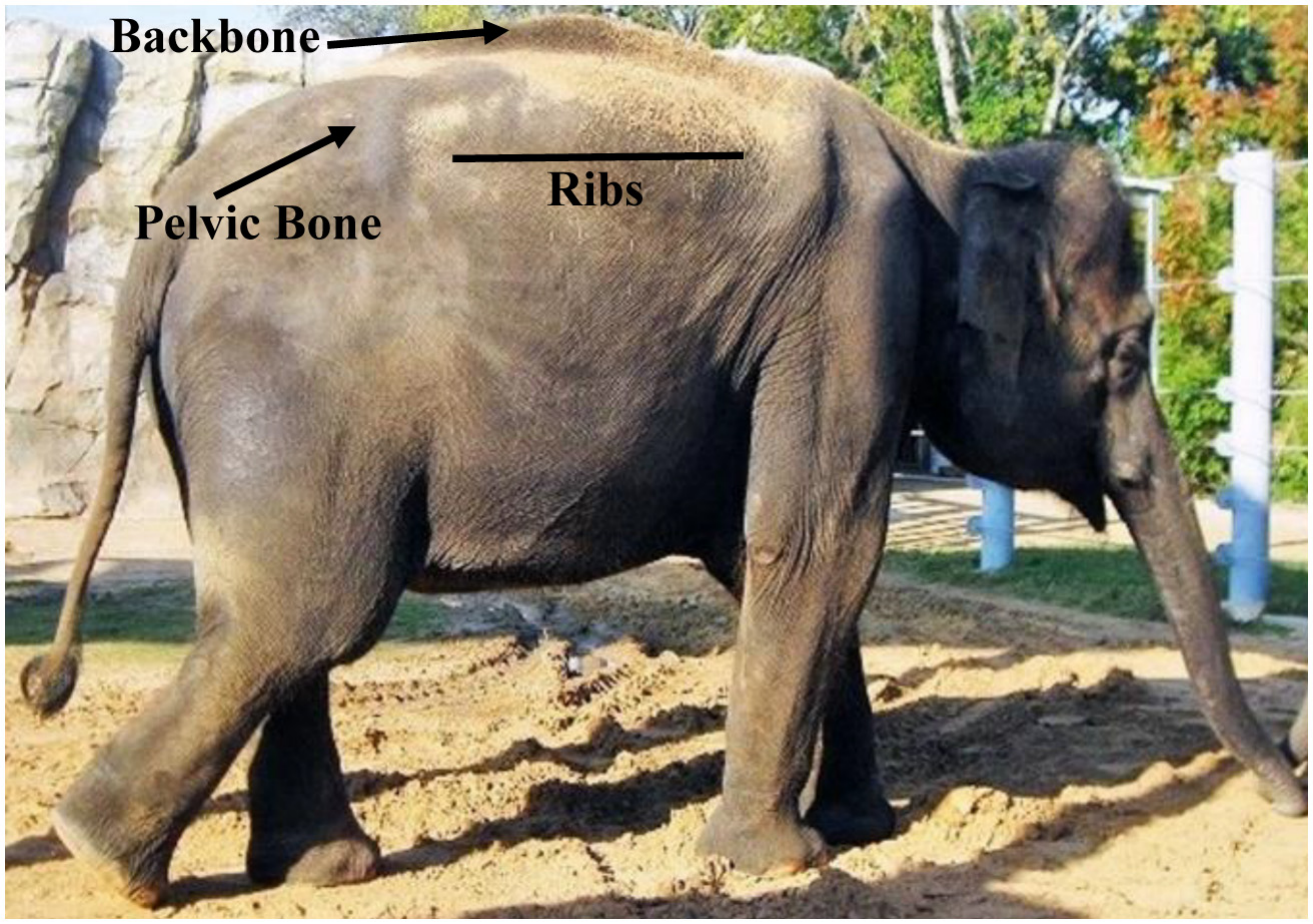
Key areas for assessing BCS in Asian elephants.

**Fig 2:**
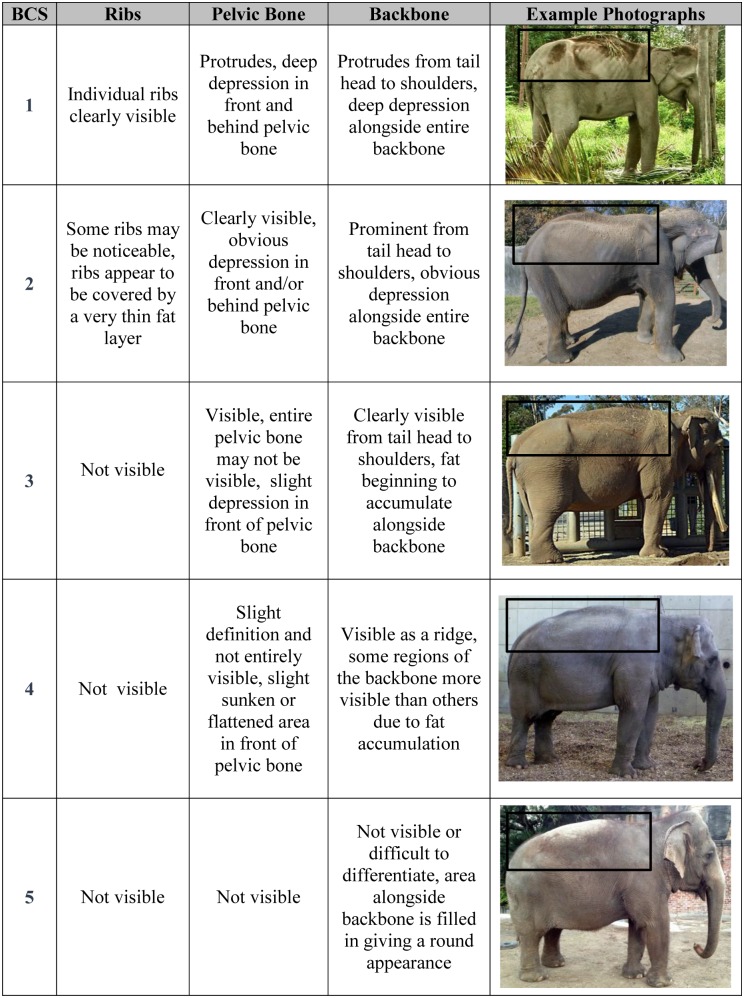
Body condition scoring (BCS) index for Asian elephants.

To develop the Asian BCS index, a variety of photographs of elephants were evaluated to identify key body areas that would serve as the anatomical regions for assessing body fat deposition patterns (ribs, pelvis, backbone: see Fig 1). Photographs of both of zoo and free-ranging Asian elephants were used to include the possible range of body conditions (thinnest to fattest) for the species. Similar to development of the African elephant BCS index [19], comparisons were made among several photographs and each body region was assessed for physical evidence that demonstrated differences in fat deposition. These differences were then categorized in order of severity such that unique physical characteristics could be assigned to each to each BCS category (see Fig 2). For example, for BCS 5, the backbone is not visible and fat fills the region alongside the entire length of the backbone, giving a round appearance. With less fat deposition, the spinous processes of the vertebra appear making the backbone visible for BCS 4. The vertebra are progressively more visible making the backbone more pronounced for BCS 3, although some fat is still visible alongside the backbone. In thin elephants (BCS 1 and 2), the depression alongside the backbone becomes obvious due to minimal fat accumulation in this region and the backbone is visible from tail head to shoulders.

To determine inter-assessor reliability of the Asian BCS index, three assessors scored 40 sets of photographs (side view, rear-angle view, and rear view) from the study population. Raters included the lead author who developed the BCS index (Assessor A), an undergraduate student majoring in Biology (Assessor B), and a pre-veterinary student (Assessor C). Assessors B and C had no prior experience in scoring body condition of any species and were blinded to the study objectives. Assessors independently scored the photographs.

For the Asian BCS index biological validation, blood samples were collected by on-site staff without anesthesia from either an ear or leg vein. Protocols requested blood draws to occur before 12 noon. Blood was maintained at ~4°C, allowed to clot at room temperature, then centrifuged at ~1500g for ~20 min. and the serum stored at -20°C or colder until analysis. Triglyceride levels were assessed using a Dimensions XP and Integrated Plus Chemistry System (Siemens Healthcare Diagnostics, Inc., New York, USA) general chemistry panel.

### Body Condition Assessment

Zoos were provided a photographic guide containing detailed instructions on how to obtain three standardized photos for each elephant for visual body condition assessment. Elephants with complete and accurate sets of photographs were included in the analysis and all photographs were anonymized and scored by K. Morfeld using the appropriate species-specific BCS index.

### Epidemiological Analysis

Independent variables were selected based on hypotheses regarding their potential association with BCS. Definitions for the independent variables selected for testing in this study are described in Table 1. Details on the collection and calculation of independent variables are presented by Meehan et al. [50], Prado-Oviedo et al. [51], and Greco et al. [52].

**Table 1:** Description of study variables for assessing associations with body condition score in Asian and African elephants.

Variable ^a^	Unit of Analysis	Description
**Demographics** ^**1**^		
Age	Elephant	Age of elephant (years)
Sex	Elephant	Female or male
Species	Elephant	African or Asian
Origin	Elephant	Captive or wild born
**Exercise** ^**2**^		
Exercise Week	Elephant	Number of reported hours spent exercising animals each week; ranging from 1 (< 1 hour per week) to 7 (14 or more hours per week)
Walk Week	Elephant	Number of reported hours spent walking elephants each week; ranging from 1 (< 1 hour per week) to 7 (14 or more hours per week)
**Feeding** ^**2**^		
Feed Day	Zoo	Number of feedings during the day
Feed Night	Zoo	Number of feedings during the night
Feed Total	Zoo	Sum of feedings during the day and night
Feeding Predictability	Zoo	The predictability of feeding activities; 1 = predictable: feeding times consistent, and may intentionally vary by up to 60 min, from day to day, and 2 = unpredictable: feeding times are not scheduled and occur randomly
Feeding Diversity	Zoo	Shannon diversity index of the number of feeding types and frequency with which each type was provided
Spread	Zoo	Relative frequency of the percentage of time food was spread around the exhibit compared to all feeding techniques
Alternative Feeding Methods	Zoo	Relative frequency of the percentage of time food was presented in a foraging device, hidden, or hanging compared to all feeding types
**Housing** ^**3**^		
Percent Time Indoor	Elephant	Percent time spent in indoor environments
Percent Time In/Out Choice	Elephant	Percent time spent in environments with an indoor/outdoor choice
Space Experience	Elephant	The average weighted (by percent time) size of all environments in which an elephant spent time.
Space Experience per Elephant	Elephant	The average weighted (by percent time) size of all environments in which an elephant spent time divided by the number of elephants sharing the environment.
**Social** ^**3**^		
Animal Contact	Elephant	Maximum number of unique elephants focal animal is in contact with
Social Group Contact	Elephant	Maximum number of unique social groups focal animal is part of
**Training and Enrichment** ^**2**^		
Rewarding Stimuli Techniques Score	Elephant	Percent time with which an elephant experienced techniques involving the provision or removal of rewarding stimuli divided by the percent time all training techniques were experienced; ranging from 1(never) to 9 (very frequently)
Enrichment Diversity	Zoo	Shannon diversity index of the number of enrichment types and frequency with which they were provided

^a^ References for variable development and description: 1. Prado-Oviedo et al. [51]; 2. Greco et al. [52]; 3. Meehan et al. [50].

Two adjustments were made to independent variables from their original format. The Space Experience variables [50] were adjusted to a value of “per 500 ft^2^” to aid in interpretation of Beta values. Feeding Predictability originally consisted of three categories [52]: “predictable” (feeding times were consistent from day to day), “semi-predictable” (feeding times were intentionally varied by up to 60 min from day to day), and “unpredictable/random” (feeding times were not scheduled or occurred randomly). To account for sample size limitations, Feeding Predictability was converted to a binary variable: predictable and semi-predictable were combined and classified as “predictable schedule” and used as the reference category in which to assess the effect of the unpredictable/random schedule.

The BCS = 3 was designated as the reference score based on the interpretation of BCS indexes used and accepted in veterinary medicine, in which the middle score represents the “ideal/normal” distribution of body fat [23–28]. This designation (BCS 3 = ideal/normal) is also utilized in the African elephant index [19], so utilizing the same scaling terminology allows for consistency across the two elephant species.

### Statistical Analysis

Frequency of body condition scores (1, 2, 3, 4, and 5) were calculated for the full population, by sex, and by species. The relationship between BCS and triglyceride levels was investigated using one-way analysis of variance (ANOVA) procedures with Scheffé’s test as a post hoc for pairwise comparisons of subgroups.

To calculate the inter-assessor reliability for the Asian BCS index, the overall percentage (%) agreement between inter-assessor assessments was calculated as (100 × *m)*/*n*, where *n* = total number of samples examined and *m* = number of cases of exact agreement. A weighted kappa (κ_*w*_) statistic was also used to analyze inter-assessor variability [53]. Standards proposed by Lanidis and Koch [54] were used to interpret resulting kappa values, where perfect agreement equates to a kappa of 1 and chance agreement equates to 0. The following standards for interpreting kappa values for strength of agreement were used: kappa values ≤ 0 = poor, 0.10 to 0.20 = slight, 0.21 to 0.40 = fair, 0.41 to 0.60 = moderate, 0.61 to 0.80 = substantial and 0.81 to 1.00 = almost perfect agreement.

A variety of demographic, management, housing, and social characteristics were evaluated as risk factors for associations with increased BCS (Table 1). Predictive models for BCS were fitted using generalized estimating equations (GEE), which allows for repeated measurement and clustering of individual animals within zoos [55–57]. Zoos were treated as random effects and an independent correlation structure was specified [57]. BCS 3 was set as the reference level and multinomial logistic regression was used to determine risk factors associated with increased BCS where BCS 4 was compared to 3 and BCS 5 was compared to 3. Multi-variable regression models were built by first assessing individual predictors at the univariate level and then at the bivariate level with demographic variables determined to be potential confounders (age, sex, species, and origin) [58, 59]. Confounding variables were included in all models and any variables that were associated with risk of increased BCS at *P* value *<*0.15 in the univariate or bivariate assessments were retained for evaluation in the hierarchical model building process.

Once a set of viable input variables and confounders was identified, the hierarchical model building process proceeded using the forward selection approach [60]. Models reaching the multi-collinearity criteria, as defined by a variance inflation factor of greater than 10 and a condition index of greater than 30, were not considered for further analysis [60]. The forward selection of variables was continued until the addition of variables no longer resulted in significant models. The final model was selected based on quasi-likelihood under the independence model criterion (QIC) values [61] and parameter estimates of explanatory variables. To aid in interpretation, Odds Ratio (OR) for assessed risk factors were calculated by exponentiation of the beta coefficients. The OR represents the ratio of the odds of an outcome (BCS 4 or 5) occurring given a particular exposure (elephant demographic or management factors) compared to the odds of the outcome occurring given non-exposure. Due to limited sample size, a similar analysis was not assessed for low BCS. Furthermore, BCS of 1 or 2 were excluded from this analysis in order to focus analysis on management-based risk factors on higher BCS, rather than lower. Statistical analyses were conducted by using SAS software, version 9.3 [PROC GENMOD, with options REPEATED, CORR = IND, DIST = MULT, LINK = CLOGIT; SAS Institute, Inc., Cary, NC]. With the exception of the univariate stage of the model building process where *P* value <0.15 was considered acceptable for continued analyses, *P* value <0.05 was considered statistically significant.

## Results

### Development and Testing of the Asian BCS Index

#### Inter-assessor Agreement

The percent agreement for assigning a BCS to the set of 40 elephants among assessors ranged from 78%-85%, with the greatest agreement between assessors A and C (Table 2). Weighted kappa values for assessments between assessors A and C were interpreted as “almost perfect” agreement, whereas all other inter-assessor agreements were interpreted as “substantial” agreement when applying the methods of Landis and Koch [54].

**Table 2:** Level of inter-assessor agreement for assessment of Asian elephant body condition. κ_*w*_ = weighted kappa; 95% CI = 95% confidence interval.

Assessors	A vs. B	A vs. C	B vs. C
**Percentage (%) agreement**	83	85	78
**κ**_***w***_ **(95% CI)**	0.78 (0.63–0.92)	0.82 (0.69–0.95)	0.70 (0.50–0.88)

#### Biological Validation

The average triglyceride levels for the BCS categories of 2–3, 4, and 5 were significantly different (Table 3). There was not a difference in the average triglyceride levels between the BCS 2 and 3 categories. The BCS 1 category was not included in the analysis due to small sample size (N = 2).

**Table 3:** Mean (SD) serum triglyceride concentrations by body condition score (BCS) category (N = 95).

BCS^1^	N	Serum triglyceride (mg/dl)	SD
1	2	13.0	2.8
2	6	24.0^a^	14.6
3	14	25.9^a^	11.2
4	31	34.5^b^	15.7
5	42	47.7^c^	21.0

^1^BCS (1 = lowest to 5 = most body fat)

^a,b,c^ Values with different letters are significantly different (*P* value<0.05)

### Body Condition Distribution

A total of 240 elephants had complete standardized sets of photographs submitted for body condition assessment. These included 108 Asian (n = 23 males, 85 females) and 132 African (n = 26 males, 106 females) elephants from 65 North American zoos.

**Fig 3:**
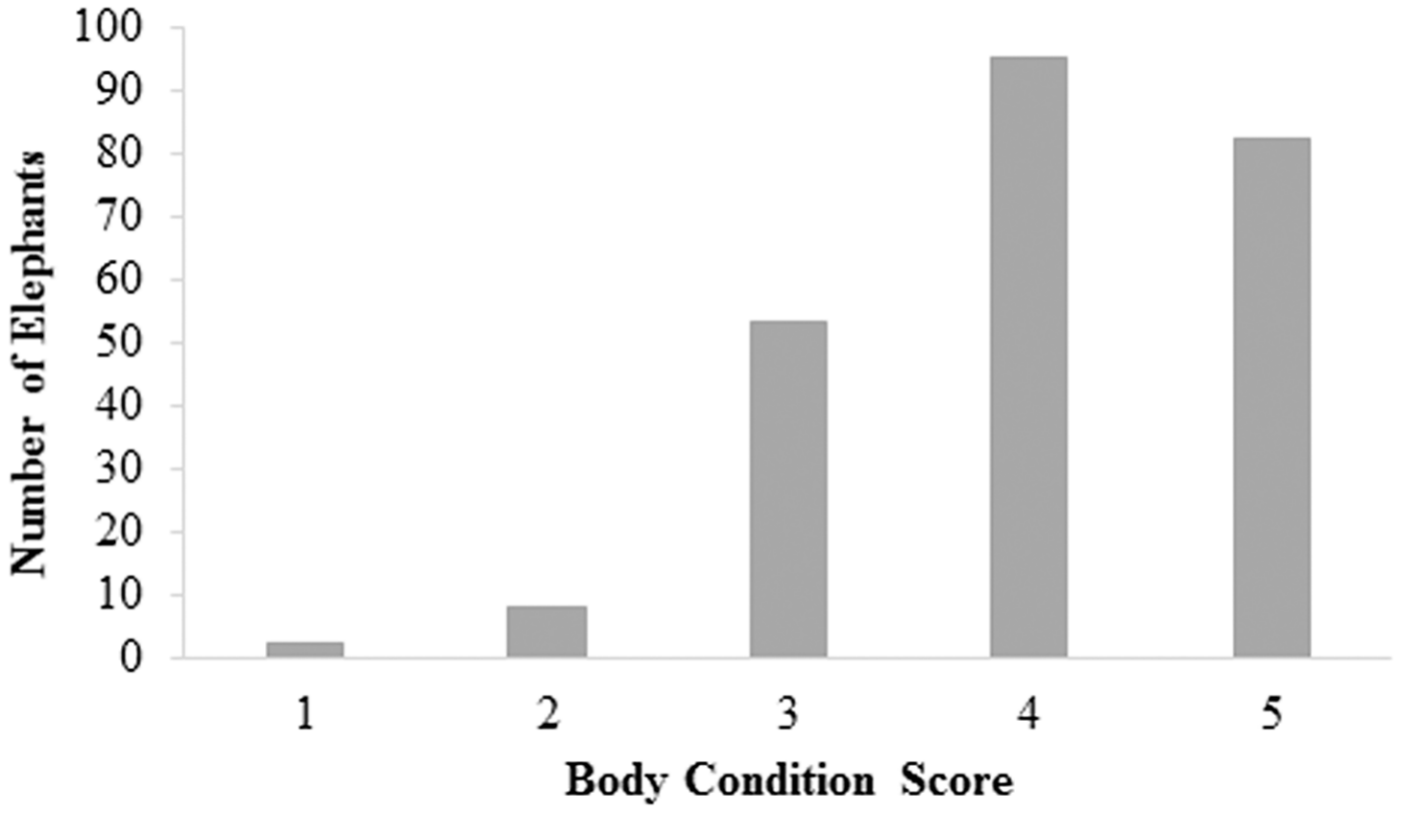
Distribution of BCS of all study elephants (n = 240, median = 4).

The distribution of BCS for the study population is shown in Fig 3 with a median BCS of 4 (n = 240, range 1–5). Collectively, only 22% (53/240) of the elephants had BCS 3. The majority of elephants had elevated BCS compared to BCS 3, with 74% of elephants in the BCS 4 (95/240; 40%) and 5 (82/240, 34%) categories. Only 10 elephants were represented in the BCS 1 (n = 2) and 2 (n = 8) categories, collectively, representing less than 5% of the study population (Fig 3).

Frequency of BCSs by species and sex is shown in Table 4. The prevalence of BCS above the reference BCS = 3, was 74% in African and 73% in Asian elephants. The most prevalent (mode) BCS observed in Asian elephants was a 5, whereas the mode in the African population was BCS 4. In males 33% had a BCS of 3, whereas 19% of females had a BCS of 3. The category representing the most body fat (i.e. obesity, BCS 5) was observed in 40% of females, whereas only 12% of males had a BCS of 5.

**Table 4:** Body condition scores for study elephants by species and sex.

	African				Asian					
	Female		Male		Female		Male		Full Population	
	N	Percent	N	Percent	N	Percent	N	Percent	N	Percent
	106		26		85		23		240	
**BCS**										
1	0	0	0	0	2	2.3	0	0	2	0.8
2	0	0	1	3.8	5	5.9	2	8.7	8	3.3
3	23	21.7	10	38.0	14	16.5	6	26.1	53	22.1
4	48	45.3	13	50.0	23	27.1	11	47.8	95	39.6
5	35	33.0	2	7.7	41	48.2	4	17.4	82	34.2

### Epidemiological Models

The results of univariate modeling of individual variables on body condition scores are presented in Table 5. Descriptive statistics for the independent variables included in the multi-variable analysis are shown in Tables 6 and 7. In the final multi-variable logistic model, the combination of Walk Week, Feeding Diversity, Feeding Predictability, and Sex had the greatest effect on risk of an elephant having a BCS of 4 or 5 compared to BCS 3 (Table 8).

**Table 5:** Independent variables tested as risk factors for BCS = 4 or 5 and statistics associated with the univariate logistic regression. OR: Odds Ratio.

Hypothesis	Variable	Reference	N	Beta	OR	*P* value
	*Demographics*					
+	Age		230	0.040	1.041	<0.001*
0	Sex	ref = Male	46			
		Female	184	1.044	2.841	<0.001*
0	Species	ref = African	131			
		Asian	99	0.579	1.784	0.077^
0	Origin	ref = Wild	168			
		Captive	58	-1.035	0.352	<0.001*
	*Exercise*					
-	Exercise Week	ref = 1	47			
		2	85	-0.598	0.549	0.193
		4	20	-0.061	0.940	0.933
		5	33	-0.566	0.567	0.187
		6	5	-1.080	0.339	0.089^
		7	15	-1.467	0.230	0.009*
-	Walk Week	ref = 1	94			
		2	71	-0.242	0.784	0.562
		4	13	-0.540	0.582	0.357
		5	12	0.078	1.081	0.902
		6	7	-0.869	0.419	0.148^
		7	8	-1.486	0.226	0.017*
	*Feeding*					
-	Feed Day		215	-0.052	0.948	0.290
-	Feed Night		215	-0.119	0.887	0.283
-	Feed Total		215	-0.047	0.953	0.223
-	Feeding Predictability	ref = 1	166			
		2	49	-0.573	0.563	0.125^
-	Feeding Diversity		215	1.262	3.533	0.038*
-	Spread		215	0.391	1.478	0.647
+	Alternative Feeding Methods		222	-0.496	0.608	0.465
	*Housing*					
+	Percent Time Indoor		228	-0.010	0.989	0.193
-	Percent Time In/Out Choice		228	0.002	1.002	0.739
-	Space Experience per Elephant		228	0.023	1.023	0.002*
-	Space Experience		228	0.005	1.005	0.009*
	*Social*					
-	Animal Contact		228	0.037	1.037	0.396
-	Social Group Contact		228	0.031	1.031	0.021*
	*Training and Enrichment*					
+	Rewarding Stimuli Techniques Score	Ref = 5	11			
		6	33	1.323	3.755	0.169
		7	89	1.710	5.531	0.077^
		8	78	1.556	4.743	0.112^
		9	2	-21.990	<0.001	<0.001*
-	Enrichment Diversity		213	-0.216	0.805	0.826

^*P* value <0.15 utilized as threshold significant level for model building

*P value <0.05. BCSs 1 and 2 are excluded from analysis.

Hypothesis: + Increase odds of having BCS 4 or 5;—Decrease odds of having BCS 4 or 5; 0 Neutral relationship

**Table 6:** Descriptive statistics for variables included in the multi-variable modeling process.

Variable	N	Mean	SD	Min	Max
Age	230	31.1	13.7	0	64
Feeding Diversity	215	1.4	0.3	0.3	1.8
Space Experience per Elephant (per 500 ft^2^)	228	22.9	24.0	0.7	201.7
Space Experience (per 500 ft^2^)	228	61.9	64.7	1.3	339.4
Social Group Contact	228	3.9	5.4	1.0	30

**Table 7:** Frequency table (count of elephants) for categorical variables included in the multi-variable modeling process.

Score	Exercise Week	Walk Week	Feeding Predictability	RSTS*
1	47	94	166	-
2	85	71	49	-
3	0	0	-	-
4	20	13	-	-
5	33	12	-	11
6	5	7	-	33
7	15	8	-	89
8	-	-	-	78
9	-	-	-	2
Total	205	205	215	213

* Rewarding Stimuli Techniques Score

**Table 8:** Multi-variable multinomial logistic regression for predicting risk for BCS = 4 or 5.

Variable	N	Beta	Odds Ratio	*P* value
Intercept 1	-	-2.1817	-	0.0813
Intercept 2	-	-0.0027	-	0.9983
Walk Week 1: Less than 1 hour per week	94	Reference	-	-
Walk Week 7: 14 or more hours per week	8	-1.552	0.212	0.011*
Feeding Diversity	215	1.546	4.692	0.008*
Feeding Predictability: Predictable	166	Reference	-	-
Feeding Predictability: Unpredictable	49	-1.175	0.309	0.007*
Sex: Male	46	Reference	-	-
Sex: Female	184	0.767	2.153	0.034*
Age	230	-0.016	0.984	0.341
Origin: Wild	168	Reference	-	-
Origin: Captive	58	0.763	2.145	0.122

*P value<0.05

Using the Odds Ratio, each of the individual variables in the multi-variable model can be explained in terms of risk for BCS 4 or 5. While Odds Ratios show the effects of each variable conditional on the other variables, it is illustrative to think about the effect each independent variable has on the probability of an outcome separately. Walk Week 7 (14 or more hours of staff-directed walking per week) was associated with BCS 4 compared to BCS 3 and with BCS 5 compared to BCS 3. In the multi-variable model, Walk Week 7 had an odds ratio of 0.212, which can be interpreted as a 78.8% decrease in odds of elephants that experience 14 or more hours of staff-directed walking per week of having a BCS above 3 as compared to elephants that experience any fewer hours of staff-directed walking per week.

**Fig 4:**
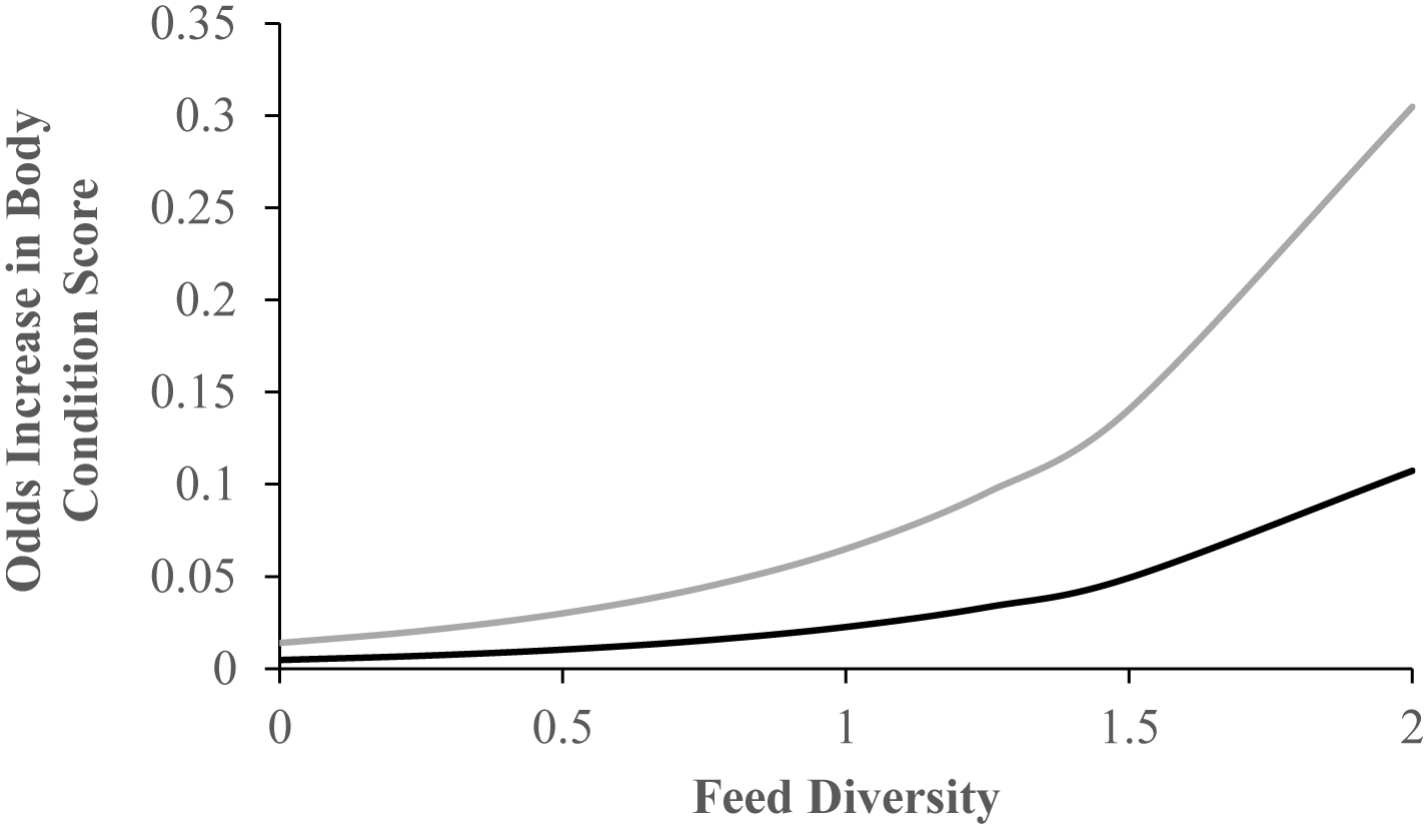
Odds increase for body condition score by Feeding Diversity for predictable (grey) and unpredictable (black) feeding schedules.

There were two feeding variables in the final model including Feeding Predictability and Feeding Diversity. The Odds Ratio for Feeding Diversity was 4.692 (Table 8). The other feeding variable in the multi-variable model, Feeding Predictability, indicates that implementing an “unpredictable or random” feeding schedule decreased the risk of BCS 4 or 5 (Table 8). Elephants that had an unpredictable feeding schedule had a 69% decreased risk of BCS 4 or 5 as compared to elephants with a predictable feeding schedule (Table 8). Fig 4 illustrates the non-linear association between Feeding Diversity and increased risk of BCS 4 or 5 for both “unpredictable” and “predictable” feeding schedules where Walk Week and Age are kept to the population averages (2 and 31.2, respectively). As the number of feeding methods and the proportion of the total feeding sessions where each method was used increased, there was an increased risk of BCS = 4 or 5, with the odds higher for predictable compared to an unpredictable feeding schedule.

Sex was a significant demographic factor, with the Odds Ratio of 2.153 indicating female elephants had a 2 fold increased risk of BCS of 4 or 5 compared to males (Table 8). In addition to being an independent variable, Sex was included as a confounder to Walk Week. Demographic variables including Age and Origin were included in the model as non-significant confounders. Age confounded Sex and Walk Week, and Origin confounded Age, Sex, Walk Week, and Feeding Diversity in the final multi-variable model.

## Discussion

This study documents the development and testing of a Body Condition Scoring index for Asian elephants as well as the assessment of body condition of zoo elephants in North America using the newly developed index as well as the companion index for African elephants [19]. Additionally, we used epidemiological methods to determine the factors associated with elevated BCS.

Our goal in developing the Asian elephant BCS index was to create a tool for assessing elephant body condition that can be utilized via assessment of standardized photographs or direct observation. In addition, we sought to improve upon a previously developed Asian elephant BCS index [62] that involves a rather extensive scoring system—six regions of the body scored using two or three criteria per region, and then the six scores are totaled to obtain an overall score ranging from 0–11 (where 0 is the thinnest). Our index reduced the number of body regions assessed from six to three based on the fact that we found no consistent visual variations in fat deposition in the head and shoulder regions. In addition, the pelvic area was included as one region in our index, while the Wemmer methods separates this area into two separate regions [62]. The Asian elephant BCS index proved to be a reliable method for assessing Asian elephant body condition based on high inter-assessor agreement across three assessors. This was also an improvement compared to the previously developed Asian elephant BCS index [62], which was not tested for reliability. Agreement can be measured using several statistics: percent agreement to provide an overall agreement rate; and the kappa statistic, which is a measure of agreement that indicates the proportion of agreement expected by chance. A weighted kappa was used to reflect the degree of disagreement so that a greater emphasis was placed on large differences between or among assessments compared to small differences. The results of the reliability testing demonstrated high percentage agreement (78% to 85%) and a “substantial” or “almost perfect” strength of agreement determined by the weighted k statistic (κ_*w*_ = 0.70 to 0.82) between assessors using the new BCS index. Our Asian and African BCS indexes can now be used to routinely assess body condition in zoo elephants, allowing for more directed management decisions to control body fat based on scientific data and validated tools.

We also sought to validate our index using a biological marker of adiposity, namely, serum triglyceride levels [33–36]. This is the first time that biological validation has been documented for an Asian elephant BCS index. While the companion African elephant BCS index was biologically validated using measures of fat thickness determined by ultrasound [19] we chose to use triglyceride levels for the validation in this study due to availability of serum samples and based on evidence from other species that serum triglycerides are accurate indicators of overall adiposity [33–36, 63]. Results from our analysis comparing triglyceride levels across body condition categories demonstrated significant differences in triglyceride levels of elephants with 3 and body conditions 4 and 5 as well as between elephants with body condition 4 as compared to 5. Therefore, the evidence indicates that the comparisons made in the multi-variable model (3 vs. 4 and 3 vs. 5) were biologically valid. There was not a significant difference in triglyceride levels between elephants of body conditions 2 and 3, indicating that perhaps visual distinctions between these two categories are not associated with differences in biological markers of adiposity. However, small sample sizes at BCS = 2 may have contributed to our inability to detect a difference that could be found if follow up studies on larger populations are conducted. As such, we can assert that there is strong evidence for biological validation of our Asian BCS index, although further research is necessary to complete this process.

Body condition scoring indexes have become integral tools in animal health management to assess the degree of fatness in a number of species. Empirically developed scales, such as our Asian and African BCS indexes, are important to elephant health management because they serve to standardize body condition assessment processes and can be used as diagnostic tools to screen for health conditions such as obesity. Often the BCS categories are interpreted to classify an individual in terms of body fat (i.e. thin, normal, or obese) with the middle score commonly representing the “ideal” or “normal” distribution of body fat [23–28]. Individuals with scores above BCS 3 are commonly classified as “overweight” (BCS 4) and “obese” (BCS 5); whereas the lower BCSs of 1 and 2 represent “thin” and “underweight” individuals, respectively. We also employed this method of classification to determine the prevalence of overweight and obese elephants in our study. However, the health implications associated with each BCS category in elephants have not been thoroughly established, so caution should be used when making health and management decisions solely based on BCS. Morfeld and Brown [64] reported that ovarian acyclicity is associated with BCS 4 and 5 and that the most prevalent BCS in reproductively cycling African elephants was a BCS of 3, which provides some evidence of functional relevance of our BCS categories. Additional investigations are certainly warranted to define what is “ideal/normal” and “obese” in terms of associated health implications.

Body condition scores for both species were generally high; the most prevalent BCS being a 4 (on a scale of 1 to 5). Across both species and sexes, the prevalence of the highest BCS 5 (suggestive of obesity) was 34%, and collectively 74% of zoo elephants were overweight (BCS 4 or 5), which is similar to rates observed in a prior study assessing body condition in zoo female African elephants [19]. The results of the multi-variable regression analysis indicated that the amount of walking-based exercise in which elephants participate is a strong predictor of BCS. This parallels findings in numerous other species, where walking has been found to be an effective means of controlling body fat [65–67]. For elephants, there appears to be a threshold below which the benefits of walking were not detected, as decreased risk of high body condition scores were observed only if the time spent in staff-directed walking was 14 hours or more per week. The method we used to assess walking activity was a survey where zoo staff provided estimates of the number of hours spent in walking-based exercise per week [52], but there could be value exploring measures of walking more quantitatively and in greater detail. We suggest future studies use motion devices like pedometers, which have successfully evaluated activity levels in a variety of species, including dogs, cattle, horses [68–70], and also elephants [71]. For example, dogs that walked more steps based on pedometer readings had better body condition [72]. Interestingly, in a parallel study that included a sub-set of our study population, Holdgate et al. [73] did not find a relationship between voluntary walking (recorded as mean daily walking distances assessed by GPS) and BCS; however, that study was not longitudinal and involved only a single measure of body condition with a 3–5 day assessment of walking distance. It is plausible that longer-term studies of elephant walking, both voluntary and staff-led, could detect associations between increasing durations of walking-based exercise and changes in body condition. It also would be worth determining the impact of walking at a higher *intensity* on body condition and associated health conditions. In humans, a higher intensity of walking decreased obesity-related mortality, whereas those walking at a very slow pace were at an increased risk [74]. Interestingly, while the amount of walking-based exercise was important to decreasing risk of high BCS in elephants, a more general exercise related variable, Exercise Week, was not. This can be explained by the fact that while “Exercise Week” was a measure of time spent in several types of staff-directed exercise, including stretching, calisthenics, intervals, strength, and swimming, of these, stretching was the most prevalent [52]. Given the lower caloric demands of stretching-based exercises, it is reasonable that elephants that spend more time stretching would not be more likely to have an “ideal/normal” BCS.

A number of feeding variables were related to body condition, including Feeding Diversity and Feeding Predictability. We originally hypothesized that a higher Feeding Diversity would predict a decreased risk of BCS 4 or 5 because more dynamic feeding programs would lead to greater activity levels as elephants moved around exhibits to access different types of feeding opportunities. Indeed, there was a significant relationship between increased Feeding Diversity and voluntary walking distance in a sub-set of the zoo elephants in our study [73]. However, this relationship did not extend to a decreased risk of high BCS [73]. One possible explanation is that when greater numbers of different feeding methods are used more frequently (increased Feeding Diversity), there may be an accompanying increase in the *quantity* of food provided/consumed as well. It is important to note that while we did account for the number of times food was presented (Feed Day, Feed Night and Feed Total) and none of these variables were associated with BCS outcomes, we did not account for *quantity* or *quality* of food provided or consumed, and so future studies should include assessments of dietary composition and total nutrient intake, factors that can be associated with body fat deposition [75].

The schedule of feedings also was important, with results indicating that the implementation of an unpredictable feeding schedule is associated with decreased risk of high body condition. In fact, elephants had a 69% decrease in risk of BCS 4 or 5 if food was provided on an unpredictable schedule throughout a 24-hour period. This finding is supported by studies in humans, in which varying feeding times and frequency reduces the risk of obesity and cardiovascular disease [75]. Furthermore, varying the *timing* of feedings is now used as a simple method for preventing obesity in people [76]. Eating multiple, small meals at varying times throughout the day rather than a few larger meals at set times may work by suppressing hunger and lowering serum insulin concentrations [77]. By contrast, a low number of predictable feedings results in higher insulin compared to high frequency, unpredictable feedings [77–81]. This relationship could lead to higher body condition as insulin inhibits lipase enzyme activity and increases fat deposition, thus resulting in excess body fat deposition under conditions of high insulin production. In elephants, Morfeld et al. [64] found elevated serum insulin concentrations in elephants with high BCSs, so a similar mechanism involving insulin regulation may contribute to excessive fat and high BCSs in zoo elephants. Given that 78% of all study elephants were on a predictable feeding schedule, implementing an unpredictable feed schedule could have a positive effect on body condition. Perhaps inclusion of automatic feeders into elephant exhibits, such as those used in the equine industry, would provide zoos a convenient way to deliver unpredictable feedings over 24 h, thus simulating the elephant’s natural foraging behaviors.

Sex was a significant factor affecting BCS, with females having a higher BCS than males. In fact, only 8% of African and 17% of Asian males were a BCS 5, whereas 48% of Asian and 33% of African females were in this category. A trend towards higher body fat in females compared to males has also been documented in humans [82], dogs [83], and horses [84]. Perhaps future studies should investigate sex adjustments in the elephant scoring systems, similar to sex-adjusted scales for humans [3]. Lower BCS in males may be related to higher energy requirements for breeding, similar to that found for stallions [85]. Musth status may also play in role in the lower BCSs in males compared to females, similar to that observed in the wild [86, 87] and is deserving of further study in zoo elephants.

Last, a training variable was associated with high BCS in the univariate analysis results, namely the frequency with which “Rewarding Stimuli Techniques” were utilized during training sessions. Although this variable was not included in the final multi-variable model, it is worth exploring given the significant role of training in elephant management. A high BCS is associated with more frequent use of techniques associated with Rewarding Stimuli- most commonly food or verbal praise- to reinforce desired behavior [52]. Therefore, it is possible that the correlation between higher RST scores and high BCS may be a reflection of excess calorie intake incurred during training interactions. Given the fact that training is an essential part of elephant management and rewards are an essential element to positive-reinforcement-based training [52], it may be advisable to investigate utilizing lower calorie treats, foods with a lower glycemic index or non-food rewards during training to decrease risk of high body condition in zoo elephants.

## Conclusion

Nearly three-quarters of the elephants in the North American zoo population were classified as having body condition suggestive of being overweight or obese. Given the important role that body condition plays in health outcomes for humans and other species, it is clear that assessing body condition effectively is an essential tool in elephant management. However, the connection between BCS and adverse health outcomes for the North American zoo elephant population are just beginning to be described. High BCS and metabolic hormones, specifically insulin and leptin, were found to be predictors of ovarian cyclicity in female African elephants [64] however no associations were found between BCS and foot health or musculoskeletal health for either species or sex in the North American zoo population [88]. Therefore, it is important to continue exploring these relationships longer-term and over a wider range of health outcomes, including cardiovascular disease and metabolic disorders. To that end, we have provided a new reliable Asian elephant BCS index that improves upon previously published tools and, in conjunction with Morfeld’s African elephant BCS index [19], can be efficiently utilized by zoo professionals for longitudinal monitoring elephant body condition. We are encouraged by evidence that exercise and feeding related management practices are related to body condition of the North American zoo elephant populations, which suggests that rather simple changes may have a significant effect on individual health and welfare.
